# Characterization of Nasal Mucosal T Cells in Horses and Their Response to Equine Herpesvirus Type 1

**DOI:** 10.3390/v16101514

**Published:** 2024-09-25

**Authors:** Camille M. Holmes, Bettina Wagner

**Affiliations:** Department of Population Medicine and Diagnostic Science, College of Veterinary Medicine, Cornell University, Ithaca, NY 14853, USA; cmh335@cornell.edu

**Keywords:** adaptive immune response, mucosal immunity, T cell, viral infection, herpesvirus

## Abstract

Equine herpesvirus type 1 (EHV-1) enters through the upper respiratory tract (URT). Mucosal immunity at the URT is crucial in limiting viral infection and morbidity. Here, intranasal immune cells were collected from horses (*n* = 15) during an experimental EHV-1 infection. CD4^+^ and CD8^+^ T cells were the major intranasal cell populations before infection and increased significantly by day six and fourteen post-infection, respectively. Nasal mucosal T cells were further characterized in healthy horses. Compared to peripheral blood mononuclear cells (PBMC), mucosal CD8^+^ T-cell percentages were elevated, while CD4^+^ T-cell percentages were similar. A small population of CD4^+^CD8^+^ T cells was also recovered from mucosal samples. Within the URT tissue, CD4^+^ cells predominantly accumulated in the epithelial layer, while most CD8^+^ cells resided deeper in the mucosa or the submucosa below the basement membrane. In vitro stimulation of mucosal cells from healthy horses with (*n* = 5) or without (*n* = 5) peripheral T-cell immunity against EHV-1 induced IFN-γ production in nasal T cells upon polyclonal stimulation. However, after EHV-1 re-stimulation, mucosal T cells failed to respond with IFN-γ. This work provided the first characterization of mucosal T-cell phenotypes and functions in the URT of healthy horses and during EHV-1 infection.

## 1. Introduction

The respiratory system faces constant environmental exposure, making it the primary entry site of many pathogens that greatly impact human and animal health. The mucosal immune system represents a distinct portion of the body’s first line of defense, with a crucial role in maintaining homeostasis at the mucosal barriers, requiring a delicate balance to maintain physiological function while simultaneously limiting pathogen infiltration. During acute respiratory viral infection, a variety of distinct T-cell populations are involved in mucosal immune responses. In humans and mice, this includes intraepithelial T cells (T-IEL), T cells from mucosal-associated lymphoid tissue (MALT) localized to the site of primary infection, and circulating peripheral T cells that can be recruited to the mucosa [1,2,3,4]. In humans and in mouse models of human disease, mucosal T cells contribute to anti-viral defense through the rapid elimination of infected cells, which reduces systemic pathogen infiltration [5,6]. The major causes of viral respiratory disease in horses are equine herpesviruses and equine influenza [7]. Equine herpesvirus type 1 (EHV-1) is particularly concerning due to its severe clinical outcomes, including abortion and equine herpesvirus myeloencephalopathy (EHM).

EHV-1 is prevalent in equine populations worldwide, infecting an estimated 80% of horses during their lifetime [8] and achieving latency in about 60% of infected animals [9,10]. Upon infection of the upper respiratory tract (URT), susceptible horses will develop respiratory disease, characterized by nasal and ocular discharge, fever, and lethargy [11,12]. Infection of nasal epithelial cells causes damage to the mucosal barrier [13], leading to local cytokine production and inflammation [14,15], and the recruitment of leukocytes. This results in viral dissemination into the lymphoid tissue [16] and the establishment of a cell-associated viremia [17]. During the latter phase, EHV-1 can spread and replicate in endothelial cells of blood vessels, which causes ischemia and damage to the affected tissues [18]. This is of particular importance in the placenta and central nervous system, where loss of oxygenation can lead to abortion or to the neurological form of EHV-1 infection, EHM, respectively [19,20,21,22,23].

At the URT, the mucosal immune response to EHV-1 is initiated by the secretion of inflammatory cytokines and chemokines from both innate immune cells and epithelial cells [14,15,24]. In susceptible horses, the type I interferon response drives the early anti-viral state [24,25], and chemokines including c-c motif ligand 2 (CCL2), CCL3, and CCL5 are produced [24,26] which, in other species, have known roles in orchestrating immune cell homing to the tissue [27]. In previously exposed horses with immunity, EHV-1-specific T cells with cytolytic capacity can be recovered from the local lymph nodes, lymphatic tissues, and lung [12,28]. Immune horses also respond with intranasal secretion of EHV-1-specific IgG4/7 antibodies [15,24], which are capable of neutralizing the virus and preventing replication at the primary site of infection [29]. In contrast, EHV-1-specific mucosal IgA antibody concentrations do not significantly increase upon infection [15,24], and mucosal IgA did not neutralize EHV-1 in vitro [29], suggesting some role of mucosal IgA in the first line of defense but only minor contributions to adaptive immune responses during EHV-1 infection [29]. URT epithelial explant models have been used to characterize viral processes at the site of entry. This model supported a mechanism by which localized immune cells, namely CD172^+^ cells and T cells, can become infected and migrate through the basement membrane, facilitating viral transmission beyond the mucosa and into the periphery [13,30,31,32]. These studies also recapitulated some findings from in vivo infection, such as the production of chemokines CCL2 and CCL5, supporting immune cell recruitment to the mucosa upon infection [30]. While explants have been useful for studying pathogenesis, including viral replication and infiltration [13,30,32,33], their isolation from the remainder of the immune system has hindered exploration of anti-viral host immunity. Thus, a protective T-cell response in the URT has not yet been characterized. 

Several studies concluded that both pre-existing EHV-1-specific antibodies in serum [24,34] and EHV-1-specific T cells in peripheral blood [10,24,35,36] correlate with protection against EHV-1 infection and disease. Following infection, previously exposed horses established detectable CD8^+^ T-cell responses by day seven post-infection (pi), whereas the response was delayed in naïve animals [37,38]. The peak of the T-cell response occurs between day 14 and 21pi [37,38], with a detectable population of circulating T cells for up to one year afterward [38]. Virally stimulated T cells can secrete IFN-γ in vitro [39,40,41,42,43], with the majority of IFN-γ-producing cells being CD8^+^ cytotoxic T cells (CTLs) early after infection [14,39,41]. A few months after infection, the EHV-1-specific IFN-γ-producing population shifts from a predominantly CD8^+^- to a CD4^+^-T-cell-dominated response [42,44,45]. The establishment and phenotype of the EHV-1-specific IFN-γ-expressing population can be dependent on the age of the horses and on how immunity was developed, either via infection or vaccination. EHV-1 infection in mice also induced an EHV-1-specific CD8^+^-T-cell response [46], and CD8^+^-T-cell depletion in these models lessens protection [47], underscoring the role of CD8^+^ T cells in EHV-1 immunity. 

Although EHV-1-specific T cells are detectable in the peripheral blood of healthy horses, mucosal immune cells at the URT and their response to EHV-1 infection have not yet been analyzed. In this study, our objective was to investigate the role of nasal mucosal T cells in the EHV-1 response, characterize the nasal T-cell populations in the URT, and assess their functional capacity.

## 2. Materials and Methods

### 2.1. Horses

All animals in these studies were from Cornell University’s herd of Icelandic horses. Two separate cohorts were used: horses experimentally infected with EHV-1 (*n* = 15) and clinically healthy horses (*n* = 10). EHV-1-infected horses were seven months of age including both fillies (*n* = 6) and colts (*n* = 9). The experimental infection was previously described in detail [45] and the clinical outcomes are summarized in Table 1. Within this cohort, some foals had received a glycoprotein vaccine at birth. However, none of the infected foals had detectable immunity against EHV-1 prior to infection at weanling age as previously described [45]. Healthy horses included a larger range of ages with a median of 10 years (range 2–19 years) including both mares (*n* = 3) and geldings (*n* = 7). Within this cohort, five horses had detectable cellular immunity from prior EHV-1 infection or vaccination with high IFN-γ responses to in vitro infection, and five horses had low IFN-γ responses (Table 2). At the time of sampling, all horses were healthy and without exposure to EHV-1 or EHV vaccination within the last 2.5 years. 

All horse procedures were approved by the Institutional Animal Care and Use Committee at Cornell University (protocol #2011−0011). Additionally, they were carried out by the recommendations in the Guide for the Care and Use of Laboratory Animals of the National Institutes of Health and the Guide for Care and Use of Animals in Agricultural Research and Teaching. 

### 2.2. Experimental EHV-1 Infection In Vivo

Horses were experimentally infected with EHV-1, as previously described [45]. Briefly, horses were intranasally infected with 1 × 10^7^ plaque-forming units of EHV-1 strain NY03 using a mucosal atomizer device (Wolfe Tory Medical, Salt Lake City, UT, USA). Physical exams were conducted including temperature, and nasal and blood samples were collected to evaluate clinical and virological status. The outcomes of infection are summarized in Table 1. All horses were susceptible to infection and developed clinical signs of respiratory disease, and the virus was detected in nasal secretion samples and peripheral blood mononuclear cells (PBMC), indicating cell-associated viremia. 

### 2.3. Nasal and Peripheral Cell Collection and Processing

Cells were collected from EHV-1-infected horses by nasal wash sampling, and from healthy horses by peripheral blood, nasal wash, nasal swab, and nasal brush sampling. Each method targeted a different cell population. Peripheral blood was collected by jugular venipuncture into tubes containing heparin, utilizing a vacutainer system (Becton Dickinson, Franklin Lakes, NJ, USA), to isolate PBMC. Intranasal cells were collected by nasal wash using 50 mL of sterile saline, which was intranasally administered with a catheter (12fr × 16inch sterile urethral catheter, Cardinal Health, Dublin, OH, USA) and recovered in a clean plastic bag positioned under the horse’s nose. Nasal mucosal surface cells were collected using two sterile, polyester-tipped swabs (Puritan Medical Products Company, Gullford, ME, USA), and nasal mucosal cells were collected by cytology brush (Puritan Medical Products Company, Gullford, ME, USA). Nasal swabs and brushes were rotated gently in the nasal cavity, contacting the mucosal surface for 5 s, and then transferred into a sterile tube containing 2 mL phosphate-buffered saline (PBS, 137 mM NaCl, 2.7 mM KCl, 4.3 mM Na_2_HPO). All samples were immediately transferred to the laboratory for processing. Nasal brushes and swabs were washed with additional PBS to dislodge cells. All nasal samples were centrifuged at 300× *g* for 5 min to pellet cells, supernatants were removed, and cells were resuspended in sterile PBS. Next, cells from all sample types were isolated by density centrifugation (Ficoll-Paque PLUS; GE Healthcare, Piscataway, NJ, USA). Isolated cells were then resuspended in DMEM medium (DMEM supplemented with 1% (*v*/*v*) nonessential amino acids, 2 mM l-glutamine, 50 µM 2-ME, 50 µg/mL gentamicin, 100 U/mL penicillin, 100 µg/mL streptomycin (Thermo Fisher Scientific Waltham, MA, USA), and 10% FCS) at 3 × 10^6^ cells/mL for use in in vitro assays. Cells utilized for ex vivo characterization were washed once in PBS, fixed in 2% formaldehyde for 20 min at room temperature, and washed twice in PBS.

### 2.4. In Vitro T Cell Stimulation

Isolated cells were incubated for a total of 48 h at 37 °C, with 3 × 10^6^ cells per aliquot. Cells were either re-stimulated with the neuropathogenic Ab4 strain of EHV-1 at an MOI of 1 or incubated in medium for 48 h. An additional cell aliquot was stimulated with Phorbol 12-myristate 13-acetate (PMA, Thermo Fisher Scientific, Waltham, MA, USA) and ionomycin (iono) (Thermo Fisher Scientific, Waltham, MA, USA) for the last 24 h of the incubation period. At 24 h, Brefeldin A (Thermo Fisher Scientific, Waltham, MA, USA) was added to all cells that were used for flow cytometry. Following incubation, cells used for flow cytometry were harvested, washed in PBS to remove medium, and fixed in formaldehyde as described above. Additionally, cell culture supernatant was harvested from wells containing 3 × 10^6^ cells/mL, which were not treated with Brefeldin A, for detection of IFN-γ secretion.

### 2.5. Intra- and Extracellular Staining for Flow Cytometry

Fixed cells were characterized by multi-color staining and flow cytometry. Ex vivo cells from EHV-1-infected horses were characterized by extracellular double staining for the leukocyte marker LFA-1 (cz3.2), [48] and either CD4 (HB61A) [49], CD8 (CVS8) [50], the regulatory T-cell marker CD25 (15-1) [51], the natural killer (NK) cell marker NCR1 (NK p46) [52], IgM (1-22) [53], IgG1 (CVS45) [50], the latter two for staining B cells, or the monocyte/macrophage marker CD14 (105) [54]. Ex vivo cells from healthy horses were characterized by extracellular staining for LFA-1, CD4, and CD8 to determine T-cell subpopulations. A minimum of 10,000 events per sample were recorded. In vitro stimulated cells were characterized after incubation by intracellular staining for IFN-γ (38-1) in saponin buffer (PBS containing 0.5% saponin, 0.5% BSA, and 0.02% NaN_3_), and extracellular staining for LFA-1 and CD4 or CD8. A minimum of 100,000 events per sample were recorded. All cells were read in a FACSCanto II flow cytometer (BD Biosciences, San Diego, CA, USA) and analyzed using FlowJo version 10.8.1 (FlowJo, Ashland, OR, USA).

### 2.6. Fluorescent Bead-Based Assay for IFN-γ

Cell culture supernatants from equine PBMC were measured in a fluorescent bead-based assay for IFN-γ secretion, as previously described [42,55]. Supernatants from cells cultured in medium provided a control to determine production of IFN-γ in the absence of stimulation, and this value was subtracted from the value measured in the Ab4 EHV-1-infected cell culture supernatant, for each horse. IFN-γ secretion values were expressed as mean fluorescence intensity (MFI).

### 2.7. Tissue Collection and Immunofluorescence

Nasal epithelial tissue was collected from the ventral nasal concha of two 12-year-old, healthy Icelandic geldings, and immediately transferred into a tube containing phosphate-buffered saline (PBS, 137 mM NaCl. 2.7 mM KCl. 4.3 mM Na_2_HPO) and transported on ice. Tissue was washed with PBS to remove any blood and immediately frozen at −80 °C in Optimal Cutting Temperature compound (Tissue-Tek OCT, Sakura Finetek USA, Inc. Torrance, CA, USA). Tissue was cut by cryostat into 10 μm sections and adhered to charged glass slides. Before staining, tissue was fixed in 10% (*v*/*v*) formalin solution (VWR, Radnor, PA, USA) and washed twice in deionized water, each for ten minutes. Then, tissue was blocked for one hour with 10% fetal calf serum (FCS (Atlanta Biologicals, Flowery Branch, GA, USA)) in PBST (PBS supplemented with 0.1% Tween 20 (VWR, Radnor, PA, USA)). Tissues were stained overnight at 4 °C with CD4 (HB61A) and CD8 (CVS8) antibodies conjugated with Alexa Fluor A647 and A488 (Thermo Fisher Scientific Waltham, MA, USA), respectively. The following day, tissues were washed twice with PBST for 10 min on an orbital shaker to remove excess stain solution, dipped in DI water, then mounted using Fluoroshield with 4′,6-diamidino-2-phenylindole (DAPI, Sigma-Aldrich, St. Louis, MO, USA). Slides were visualized on a Leica DM2500 upright microscope with a DFC7000T camera for fluorescence and bright field microscopy (Leica Microsystems, Wetzlar, Germany) at 620 nm for CD4, 488 nm for CD8, and 359 nm for DAPI. Images from each wavelength were merged using Leica Application Suite X (v1.4.5, Leica Microsystems, Wetzlar, Germany).

### 2.8. Statistical Analysis

All statistical analysis was performed using GraphPad Prism software version 8 (GraphPad Software, La Jolla, CA, USA). Data sets were tested by Shapiro–Wilk normality tests, were not normally distributed, and were further analyzed using non-Gaussian statistical tests. Changes in T-cell percentages after EHV-1 infection were compared by Friedman test with Dunn’s multiple comparisons. Mucosal and systemic T-cell percentages were compared by Friedman test with Dunn’s multiple comparisons. Differences in IFN-γ production following stimulation with PMA/iono or EHV-1 were compared by two-way repeated measures ANOVA with Tukey’s multiple comparisons. Differences in IFN-γ secretion were compared by Mann–Whitney tests. All graphs were made with GraphPad Prism software version 8. 

## 3. Results

### 3.1. EHV-1 Infection Provokes an Increase in Intranasal T Cells

**Figure 1:**
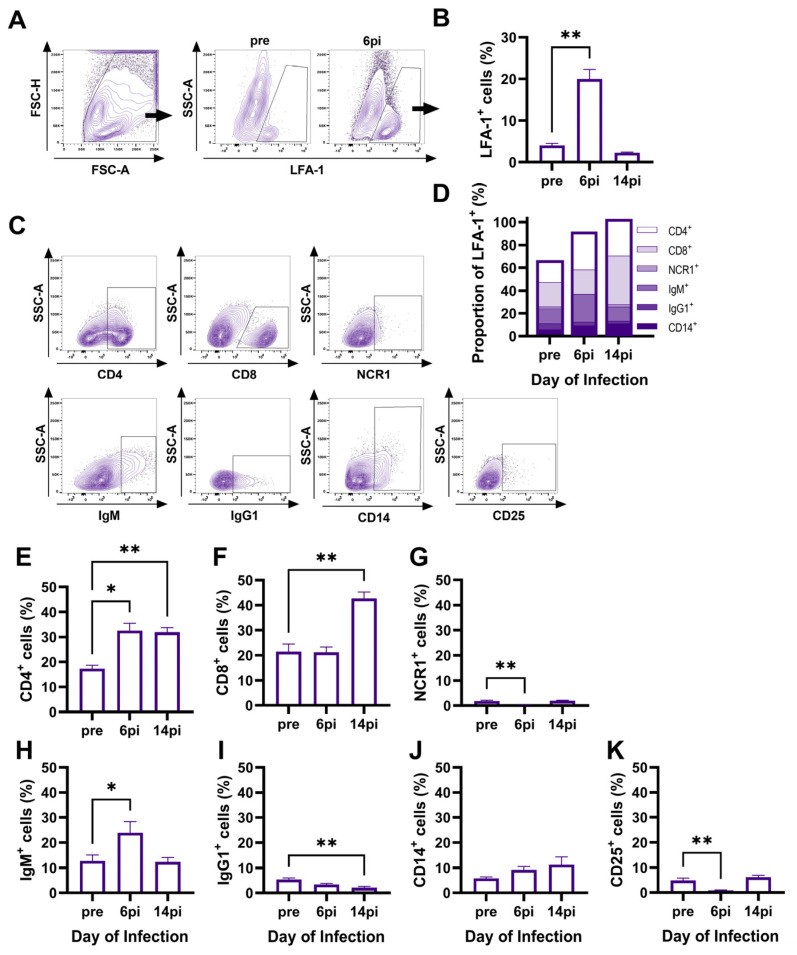
Phenotyping of mucosal immune cells during EHV-1 infection by flow cytometry. Cells were obtained from nasal wash samples (intranasal) of horses (*n* = 15) during an experimental EHV-1 infection study prior to infection (pre) and on days 6 and 14 post-infection (pi). Cells were double stained for surface expression of LFA-1 to identify immune cells, together with CD4, CD8, NCR1, IgM, IgG1, CD14, and CD25, and analyzed via flow cytometry. (**A**) A representative gating strategy from one horse is shown. Cells were gated on singlets, then on morphology, and next on LFA-1. (**B**) Percentages of LFA-1^+^ cells within the total intranasal cell population. (**C**) The LFA-1^+^ immune cell population was phenotyped using cell-type-specific markers. Plots show representative examples from one horse. (**D**) Proportions of phenotyped immune cells within the LFA-1^+^ cells. The bars give the means of each cell population for all 15 horses at each time point. (**E**–**K**) Quantification of (**E**) CD4^+^, (**F**) CD8^+^, (**G**) NCR1^+^, (**H**) IgG1^+^, (**I**) IgM^+^, (**J**) CD14^+^, and (**K**) CD25^+^ populations within the total intranasal LFA-1^+^ population pre-EHV-1 infection and on days 6 and 14pi. (**B**,**D**–**K**) Bars represent mean and (**B**,**E**–**K**) error bars represent SEM. * *p* < 0.05, ** *p* < 0.01.

Intranasal cells were collected from nasal washes of horses (*n* = 15) before (pre) and after EHV-1 infection, on days 6pi and 14pi. Cells were double stained for cell surface expression of LFA-1, to identify immune cells, and for CD4, CD8, NCR1, IgM, IgG1, CD14, and CD25 to phenotype the major immune cell populations, and were then characterized by flow cytometry. Before infection, 4.0 ± 0.5% (mean ± SEM) of intranasal cells were LFA-1^+^ immune cells (Figure 1A,B). The phenotypic characterization identified T cells as the major intranasal immune cell population, amongst IgM^+^ and IgG1^+^ B cells, CD14^+^ cells, and a few NCR1^+^ cells (Figure 1C,D). Within the pre-infection immune cell population, 17.3 ± 1.3% were CD4^+^ T cells, 21.5 ± 3.1% were CD8^+^ T cells, 1.8 ± 0.3% were NCR1^+^ NK cells, 12.8 ± 2.3% were IgM^+^ B cells, 5.4 ± 0.7% were IgG1^+^ B cells, 5.9 ± 0.6% were CD14^+^ monocytes, and 4.9 ± 0.9% of immune cells expressed cell surface CD25 (Figure 1E–K). In total, the identified immune cell subsets accounted for 64.6 ± 3.0% of the LFA-1^+^ immune cells pre-infection (Figure 1D).

Following infection, LFA1^+^ immune cell percentages increased by day 6pi (*p* = 0.002) and returned to pre-infection percentages by day 14pi (Figure 1B). On days 6 and 14pi, about 100% of the immune cells were identified with the six cell surface markers used for phenotyping (Figure 1D). In addition, EHV-1 infection resulted in unique dynamics for each cell type. CD4^+^ T cells increased by day 6pi (*p* = 0.01), which was maintained until day 14pi (*p* = 0.006) (Figure 1E). CD8^+^ T cells increased later during infection with a higher percentage on day 14pi (*p* = 0.002) compared to pre-infection (Figure 1F). NCR1^+^ NK cells represented only a small proportion of the total intranasal immune cells, decreased by day 6pi (*p* = 0.006), and returned to pre-infection percentages by day 14pi (Figure 1G). IgM^+^ B cell percentages increased on d6pi (*p* = 0.02) and then returned to pre-infection percentages by d14pi (Figure 1H). IgG1^+^ cells had a different trend, with decreasing percentages throughout infection, which was significant by day 14pi (*p* = 0.002) (Figure 1I). CD14^+^ cells did not significantly change after EHV-1 infection (Figure 1J). Finally, CD25^+^ cells decreased by d6pi (*p* = 0.006) and returned to pre-infection percentages by d14pi (Figure 1K). The differences in intranasal immune cell phenotypes between pre-infection and day 14pi supported a shift in these mucosal cell populations post-infection. The differences were mainly attributed to the increased proportions of intranasal CD4^+^ and CD8^+^ T cells, which were maintained even after total immune cell numbers decreased by d14pi, suggesting proliferation and maturation of these cells in response to EHV-1 infection.

### 3.2. Mucosal and Peripheral T-Cell Percentages in Healthy Horses

**Figure 2:**
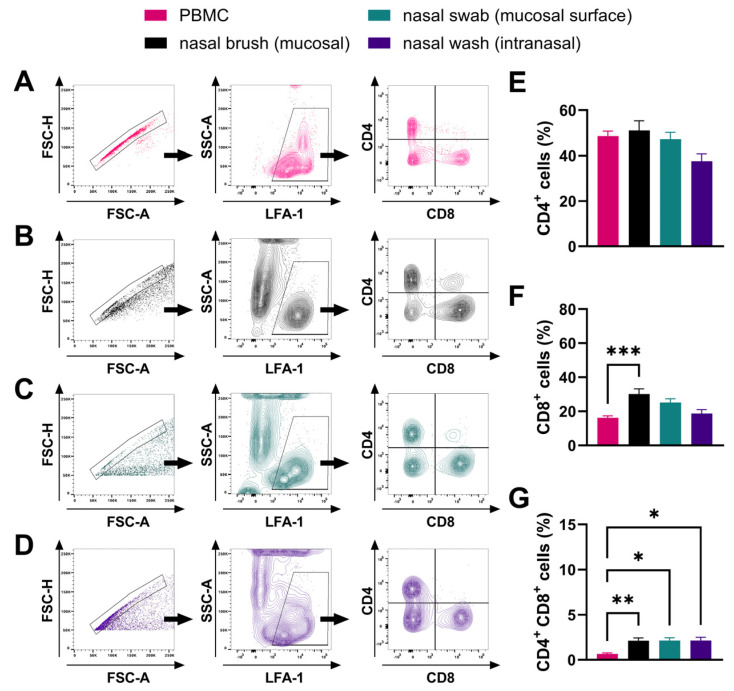
Comparison of peripheral blood and nasal mucosal T cells in healthy horses by flow cytometry. Cells were isolated from healthy horses (*n* = 10) and analyzed for surface expression of LFA-1, CD4, and CD8 by flow cytometry. Cells were isolated from (**A**) peripheral blood (PBMC, pink), (**B**) nasal brush samples (mucosal, black), (**C**) nasal swab samples (mucosal surface, green), and (**D**) nasal wash samples (intranasal, purple). Nasal brush and swab samples captured cells associated with the mucosal epithelium and nasal wash samples captured cells in the nasal lumen. A representative gating strategy for each sample type from one horse is shown. (**A**–**D**) Cells were gated on singlets, then on LFA-1^+^ cells, and finally on CD4 and CD8. Quantification of the (**E**) CD4^+^, (**F**) CD8^+^, and (**G**) CD4^+^ CD8^+^ populations within the total LFA-1^+^ immune cell population in the different sample types is displayed. (**E**–**G**) Bars represent mean and error bars represent SEM. * *p* < 0.05, ** *p* < 0.01, *** *p* < 0.001.

To further characterize nasal mucosal T cells in horses, three different procedures were compared for the collection of nasal immune cells from healthy adult horses: nasal brush, nasal swab, and nasal wash. These approaches targeted different depths in the tissue. Intranasal cells from the nasal lumen were obtained by nasal washes. Cells situated at the mucosal surface or located within the nasal mucosa were captured by nasal swab and nasal brush samples, respectively. Mucosal cell populations were compared to PBMC isolated from the peripheral blood of the same horses. Flow cytometric analysis characterized cell surface expression of CD4 and CD8 within the LFA-1^+^ immune cell population (Figure 2A–D). All samples had similar CD4^+^ T-cell percentages as in PBMC, with a trend towards a lower percentage of CD4^+^ T cells recovered from the intranasal wash samples (Figure 2E). However, CD8^+^ T-cell percentages were increased in the deeper nasal mucosa (*p* = 0.0004) samples obtained by nasal brushes (Figure 2F). Furthermore, significantly higher percentages of CD4^+^CD8^+^ (DP) T cells were observed in all nasal samples compared to PBMC. Within the total immune cell population, 2.2 ± 1.0% of mucosal (*p* = 0.006), 2.5 ± 1.0% of mucosal surface (*p* = 0.02), and 2.3 ± 1.1% of intranasal (*p* = 0.03) cells were DP T cells (Figure 2G). This demonstrated a successful recovery of mucosal T cells from healthy horses, highlighting an increase in CD8^+^ T-cell percentages in the deeper mucosal tissue, and elevated mucosal DP T cells within the URT. 

### 3.3. Location of Mucosal T Cells in the URT

**Figure 3:**
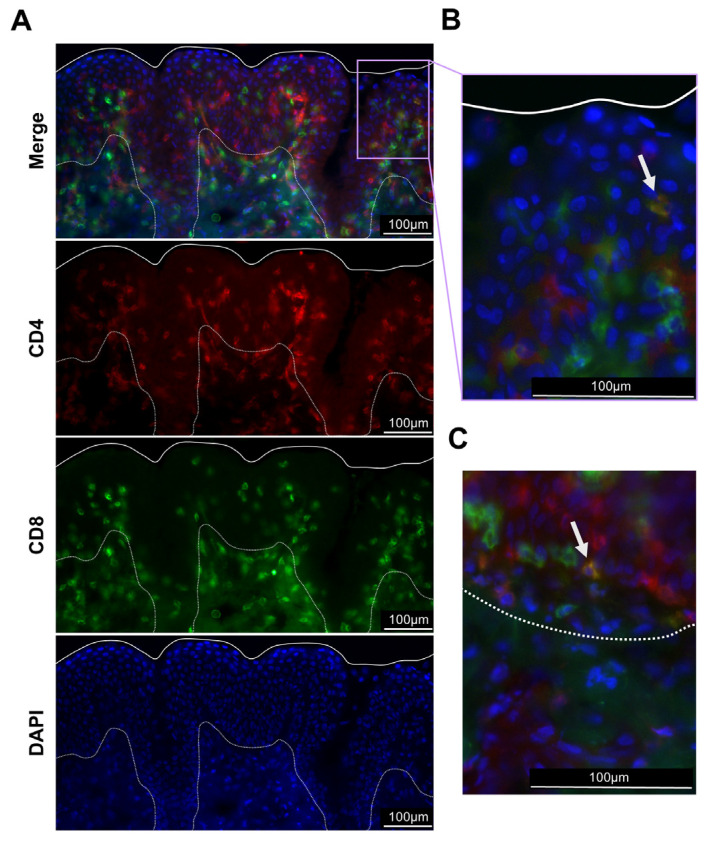
Immunofluorescence imaging of nasal mucosal tissues. To determine localization of mucosal T cells, nasal tissue samples were collected from a healthy horse and immediately frozen in OCT. Tissues were stained for CD4 (red), CD8 (green), and nuclear regions by DAPI (blue). Displayed are representative images taken at (**A**) 20× magnification and (**B**,**C**) 40× magnification. Solid white lines mark the surface of the epithelium and dotted white lines mark the basement membrane. (**B**) Displays the same field of view at different magnification of the area outlined in (**A**) in purple. (**C**) Displays a different region of the same tissue section at the basal area of the epithelium. White arrows denote CD4^+^CD8^+^ cells showing colocalization of CD4 and CD8 on the surface of one cell.

Mucosal T cells at the URT were visualized in nasal epithelial tissue of a healthy horse by immunofluorescence. Both CD4^+^ and CD8^+^ T cells were distributed throughout the epithelial layer and underlying tissue (Figure 3A). However, CD4^+^ T cells were predominantly located at the luminal site between the epithelial surface and the basement membrane, while more CD8^+^ T cells resided underneath the basement membrane in the lamina propria and the underlying submucosa. Additionally, DP T cells were present, both near the epithelial surface (Figure 3B) and the basement membrane (Figure 3C). The approach provided a spatial resolution of T-cell distribution within the nasal mucosa and was in line with the increased percentages of CD8^+^ T cells recovered by nasal brush samples, which targeted deeper regions in the tissue. Overall, immunofluorescence staining revealed high numbers of T-IEL in the nasal mucosa of healthy horses, and their non-focal, even distribution suggested a resting role of these cells, ready to become quickly activated once pathogens invade the URT.

### 3.4. Polyclonal Activation of Nasal Mucosal T Cells

**Figure 4:**
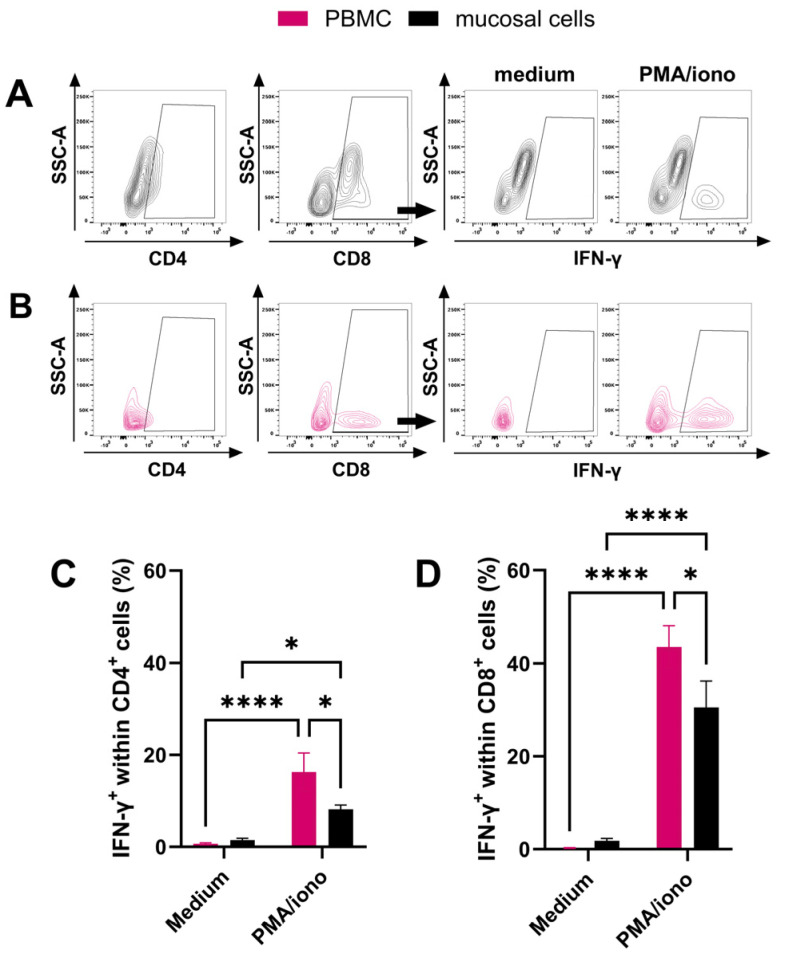
Comparison of IFN-γ production by in vitro stimulation of PBMC or mucosal cells via intracellular staining and flow cytometry. Cells were isolated from peripheral blood (PBMC, pink) and nasal brush samples (mucosal, black) of healthy horses (*n* = 10) and stimulated in vitro with PMA and ionomycin (PMA/iono). Cells were triple stained for LFA-1, IFN-γ, and CD4 or CD8, and analyzed by flow cytometry. Gates were set on singlets, then on LFA-1^+^ cells, then on CD4^+^ or CD8^+^, and finally on IFN-γ^+^ cells. A representative IFN-γ gate for (**A**) mucosal cells and (**B**) PBMC for each condition from one horse is shown. Quantification of IFN-γ^+^ cells within the (**C**) CD4^+^ or (**D**) CD8^+^ cells of ten horses is displayed. (**C**,**D**) Bars represent means and error bars represent SEM. * *p* < 0.05, **** *p* < 0.0001.

Next, the functional capacities of mucosal T cells were evaluated in comparison to PBMC. Mucosal cells collected by nasal brush were used for in vitro stimulation due to the high cell yields from this sampling procedure and to obtain T cells from the superficial and deeper tissue layers of the URT. Peripheral and mucosal cells were stimulated with PMA/iono for 24 h in vitro and compared to un-stimulated cells. Flow cytometric analysis was used to quantify IFN-γ-producing CD4^+^ and CD8^+^ T cells (Figure 4A,B). PMA/iono stimulation significantly increased expression of IFN-γ within both peripheral (*p* < 0.0001) and mucosal CD4^+^ T cells (*p* < 0.04) compared to the medium control (Figure 4C). Similarly, CD8^+^ T cells from PBMC (*p* < 0.0001) and the nasal mucosa (*p* < 0.0001) increased expression of IFN-γ during PMA/iono stimulation (Figure 4D). This confirmed that CD4^+^ and CD8^+^ nasal mucosal T cells can be activated to produce IFN-γ after polyclonal T-cell receptor (TCR) stimulation with PMA/iono. Within PMA/iono-stimulated PBMC, 16.3 ± 5.7% of CD4^+^ T cells and 43.4 ± 4.6% of CD8^+^ T cells expressed IFN-γ. Within nasal mucosal samples, 8.2 ± 0.9% of CD4^+^ T cells and 30.5 ± 5.7% of CD8^+^ T cells expressed IFN-γ. The higher percentage of IFN-γ-producing T cells in PBMC compared to mucosal T cells, within both CD4^+^ (*p* = 0.01) and CD8^+^ (*p* = 0.02) T cells, implied a lower responsiveness of the mucosal T-cell population to polyclonal stimulation through their TCR.

### 3.5. IFN-γ Responses of Mucosal T Cells after In Vitro Re-Stimulation with EHV-1

**Figure 5:**
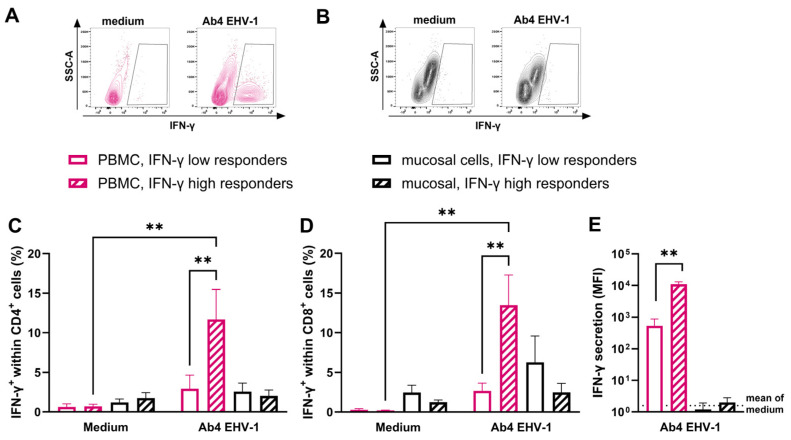
Comparison of IFN-γ^+^ cells and secretion after in vitro EHV-1 re-stimulation of PBMC and mucosal cells. Cells were isolated from peripheral blood (PBMC, pink) and nasal brush samples (mucosal, black) of healthy horses. Horses were previously categorized as IFN-γ high responders based on their response to in vitro EHV-1 re-stimulation of their PBMC (*n* = 5; striped bars), while other horses were IFN-γ low responders (*n* = 5; unfilled bars). Cell cultures were infected with the Ab4 strain of EHV-1 at an MOI of 1. Cells were stained for LFA-1, IFN-γ, and CD4 or CD8, and analyzed by flow cytometry. (**A**) PBMC and (**B**) mucosal cells were gated on singlets, then on LFA-1^+^ cells, then on CD4^+^ or CD8^+^, and (**A**,**B**) finally on IFN-γ^+^ cells. Percentages of IFN-γ^+^ cells within the PBMC and mucosal cell (**C**) CD4^+^ or (**D**) CD8^+^ population in IFN-γ low- and high-responder horses are displayed. (**E**) In addition, IFN-γ was measured in cell culture supernatants from in vitro EHV-1-infected cells. IFN-γ secretion was detected using a fluorescent bead-based assay and is shown as median fluorescent intensity (MFI) for cells of IFN-γ low and high responders. The dotted line displays the mean of MFI for uninfected cells. (**C**–**E**) Bars represent mean and SEM. ** *p* < 0.01.

To assess EHV-1-specific T-cell responses, PBMC and mucosal cells were re-stimulated with EHV-1 for 48 h in vitro. Horses were categorized into two groups that were established prior to this approach based on their PBMC’s ability to secrete IFN-γ in response to EHV-1 re-stimulation in vitro: (1) horses with high IFN-γ responses and (2) horses with low IFN-γ responses (Table 2). Flow cytometric analysis was used to quantify EHV-1-specific IFN-γ producing CD4^+^ and CD8^+^ T cells in vitro (Figure 5A,B). PBMC from IFN-γ high responders increased expression of IFN-γ within both CD4^+^ (*p* = 0.006) and CD8^+^ (*p* = 0.003) T cells after incubation with EHV-1 compared to cells in medium, while IFN-γ low-responder horses showed no difference to the medium control (Figure 5C,D). When comparing PBMC from IFN-γ high- and low-responder horses, increased percentages of EHV-1-specific IFN-γ-producing CD4^+^ (*p* = 0.009) and CD8^+^ (*p* = 0.007) T cells were observed in the high responder group (Figure 5C,D). In PBMC, 11.7 ± 8.4% of the CD4^+^ population in IFN-γ high responders were IFN-γ^+^, while only 2.9 ± 3.9% of CD4^+^ T cells expressed IFN-γ in IFN-γ low responders. Similarly, IFN-γ was expressed by 13.5 ± 8.5% of CD8^+^ T cells from IFN-γ high responders and 2.7 ± 2.2% from IFN-γ low responders. Additionally, PBMC from IFN-γ high responders secreted significantly more IFN-γ during in vitro re-stimulation (*p* = 0.008) than IFN-γ low responders (Figure 5E). In contrast, mucosal T cells in both groups had low intracellular IFN-γ expression and failed to increase secretion of IFN-γ after EHV-1 re-stimulation in vitro (Figure 5C–E). Mucosal CD8^+^ T cells from some of the IFN-γ low responders expressed IFN-γ in vitro, but there was not a significant increase between un-stimulated and EHV-1 re-stimulated cells (Figure 5D). In conclusion, nasal mucosal T cells of healthy horses did not respond to EHV-1 re-stimulation, despite an existing population of peripheral EHV-1-specific T cells in the IFN-γ high responder horses.

## 4. Discussion

The mucosal surface of the URT is the primary entry site for EHV-1. Local host immunity at the URT can majorly influence the outcome of infection and disease severity. Nasal mucosal immune responses against EHV-1 were recently explored, confirming the induction of several soluble immune proteins, including antibodies, cytokines, and chemokines, that are secreted at the viral entry site [14,15,24,34]. Striking differences in timing and pattern of local immune parameters were discovered after EHV-1 infection and clearly distinguish horses with existing EHV-1 immunity from those that are not immune and are susceptible to infection. For example, immune horses secrete high amounts of EHV-1-specific IgG4/7 mucosal antibodies within 1–2 days pi, but lack an inflammatory cytokine response at the URT [24,34]. The latter is prevented by the strong neutralizing capacity of EHV-1-specific IgG4/7 antibodies, which rapidly interfere with viral entry at the URT, disable complete viral replication cycles in the nasal epithelium, and thereby prevent EHV-1 from spreading to the peripheral blood via cell-associated viremia [24,29]. In contrast, non-immune horses produce an array of intranasal inflammatory cytokines, particularly IFN-α, CCL2, CCL3, and soluble CD14, after EHV-1 infection [14,15,24,25,34]. Both immune and non-immune horses secrete CCL5, an important chemotactic factor for homing of T cells and other immune cells [24,26], anti-inflammatory IL-10 (14,24), and the homeostatic regulator antileukoproteinase (SLPI) [25]. The latter three regulatory proteins are quickly secreted to the nasal cavity of immune horses after EHV-1 infection, while their expression in susceptible horses is delayed towards the nasal clearance phase of the virus [24,25].

In contrast, T cells in the equine URT and/or during EHV-1 infection have not previously been characterized and their phenotypes and functional capacity within the URT mucosa are mostly unknown. Work on EHV-1-specific T cells has focused on equine PBMC and supported the important role of T cells in EHV-1 immunity [10,24,35,36,42]. Both peripheral CTL and T helper (Th) cell responses have been described. While peripheral EHV-1-specific CTLs were considered crucial in the control of cell-associated viremia and thereby preventing the spread of the virus to endothelial cells [10,35,36], systemic EHV-1-specific Th1 responses are orchestrating the immune response and antibody production against the virus [42]. 

Here, we characterized for the first time nasal mucosal T cells before and after EHV-1 infection and also in healthy horses. We refined methods to recover mucosal cells from the URT, defined their location within the tissue, and determined their responsiveness to polyclonal stimulation and EHV-1 in vitro. Our current findings highlight that consistent baseline numbers of mucosal immune cells provide a robust first line of defense in healthy horses and are well positioned with-in the URT’s epithelial and other mucosal layers for rapid responses to pathogens entering the URT. Previous studies have characterized URT immune cells in equine MALT by histology, immunohistochemistry, and electron microscopy [56,57,58]. Within one MALT site, the nasopharyngeal tonsil, CD4^+^ and CD8^+^ T cells, and B cells were identified within the lymphatic structure and surrounding epithelial tissue [57]. While previous assessments focused on organized lymphoid tissues, we characterized immune cells in the nasal lumen and mucosa by comparing intranasal immune cells (nasal wash), and immune cells within the URT mucosal tissue (nasal swab or brush), in healthy horses and in horses after EHV-1 infection. Intranasal cells collected before EHV-1 infection were composed of predominantly CD4^+^ and CD8^+^ T cells, and also NK cells, B cells, and monocytes. IL-2 receptor CD25 is expressed by regulatory T cells (Tregs) and some other T cell subsets [51]. CD25 was detected in a small portion of the intranasal immune cell population. The low numbers of mucosal cells prevented further characterization of the regulatory T-cell population, warranting future study. Intranasal T cells represented nearly 40–60% of the total intranasal immune cell population in healthy horses. Especially, intranasal CD4^+^ T-cell percentages, with about 17% pre-infection, were much lower in the younger, naïve weanlings, which were used for the initial characterization of mucosal immune cells after EHV-1 infection. Healthy adult horses with prior EHV exposure had around 38% CD4^+^ T cells within their total intranasal immune cell population. However, the proportions of CD8^+^ mucosal cells were comparable between young and adult horses with around 21% and 19%, respectively. The age-related difference in these healthy horses suggests that resident adaptive CD4^+^ T-cell numbers increase with continual antigen exposure throughout life. Further characterization of mucosal cells located within the nasal epithelium also identified CD4^+^ and CD8^+^ T cells, with increasing percentages of CD8^+^ T cells in the deeper location of the mucosa. Visualization of nasal mucosal tissue emphasized the wide spread of T cells in the URT mucosa and preferences in the spatial distribution of T cells, with CD8^+^ T cells situated closer to the basement membrane and CD4^+^ T cells located closer to the lumen. 

The recovery of T cells from the mucosa of healthy horses and their visualization by immunofluorescence confirmed the presence of mucosal and intraepithelial T cells (T-IELs). In humans, T-IELs represent a large proportion of cells within the epithelial surface, with an estimated ratio of one T-IEL to ten epithelial cells [1]. Beyond CD4 and CD8 expression, several distinct T-IEL phenotypes have been characterized in mice and humans and can broadly be defined as conventional or unconventional T cells. Conventional T-IELs undergo post-thymic activation in the presence of foreign antigens and act as adaptive immune cells within the tissue environment, where they can establish as tissue-resident memory T (T_RM_) cells at the site of primary infection [2,59,60,61,62,63]. Additionally, human and murine resident Tregs play a crucial role in maintaining homeostasis by promoting appropriate effector responses and limiting inflammation during antigen exposure [64]. Unconventional T-IELs are more innate-like and many different populations exist, which can be further divided based on TCR usage: (1) invariant TCRs, including mucosal-associated invariable T (MAIT) cells, or (2) diverse TCRs, including unconventional αβ T cells and γδ T cells. Antigen specificity of MAIT cells is restricted to small molecule metabolites [65]. Unconventional αβ T cells and γδ T cells have a broader range of recognition including bacterial and self-derived antigens [66]. Unconventional T cells play crucial roles in barrier homeostasis and can respond to pathogens by the production of inflammatory cytokines and release of cytotoxic granules [67,68]. The population of nasal mucosal T cells from healthy horses readily responded to PMA/iono stimulation with IFN-γ production, which is consistent with findings in human T_RM_ and MAIT cells [69,70]. PMA/iono activates intracellular T-cell pathways in a TCR-independent manner, resulting in the production and secretion of cytokines and cytotoxic granules. During normal TCR engagement, protein kinase C activity and Ca^2+^ influx, directly downstream of the TCR, induce a signaling cascade to promote T-cell gene expression. PMA and iono mimic TCR signaling through activation of protein kinase C and regulation of Ca^2+^ channels to increase intracellular Ca^2+^ levels, respectively. Together, this treatment bypasses the TCR, allowing for polyclonal stimulation of T cells to determine their effector capacity, regardless of antigen specificity. In this study, we utilized CD4 and CD8 expression to identify T-cell subpopulations. While murine T_RM_, MAIT, and unconventional αβ T-IELs can express one or both co-receptors, most γδ T cells lack expression of either [68,71]. In horses, markers for γδ T cells are missing, and additional phenotyping of mucosal double negative T cells by gene expression is needed in future studies. With the available phenotypic markers, we lacked characterizing about 20-40% of mucosal immune cells in healthy horses. These not-yet characterized cells could represent equine mucosal γδ T cells in the URT of healthy horses. 

The use of multi-color flow cytometry and immunofluorescence also identified mucosal DP T cells for the first time in the equine URT mucosa. While historically viewed as a developmental stage, the presence of extra-thymic DP T cells supports their functional role in mucosal immunity. The regulation of CD4 versus CD8 fate in the thymus is mediated by expression of several transcription factors, [72,73,74]; however, in mice and humans, there is in vitro and in vivo evidence for post-thymic transcriptional reprogramming of CD4^+^ and CD8^+^ T cells, resulting in mucosal DP T cells [72,75,76,77]. DP T cells have also been identified in swine peripheral blood at high percentages, and in lower percentages in human and mouse peripheral blood, which aligns more closely with DP T cells in horses [78]. Additionally, mucosal DP T cells are abundant in the swine and human digestive tracts, at higher percentages compared to the periphery [79,80]. While mucosal DP T cells in the respiratory tract are largely unexplored, the high percentage described here in horses agrees with what has been observed in the digestive tract mucosa. In humans, DP T cells can act as functional immune cells through production of IFN-γ and cytotoxic effector molecules, supporting their relevance in immunity [72,81]. The isolation techniques employed in this study were designed to limit invasiveness, and the overall low number of DP T cells prevented further characterization using these methods, warranting further investigation in future studies.

Overall, our analysis of nasal mucosal T cells in healthy horses showed that they are ubiquitous and in a prime location for rapid responses to infectious pathogens, such as EHV-1. While immune cell numbers in the lumen of the URT were low in healthy horses, we identified an influx of LFA-1^+^ intranasal immune cells by day 6 after experimental EHV-1 infection. CD4^+^ T-cell proportions increased by d6pi. In healthy horses, CD4^+^ T cells were positioned closer to the surface of the epithelium, likely allowing for more immediate antigen recognition, activation, and proliferation. By day 14pi, the total number of immune cells had decreased to pre-infection values, but CD4^+^ and CD8^+^ T-cell percentages remained significantly higher than pre-infection. This shift in the proportions of intranasal immune cells was combined with the disappearance of non-characterized immune cells by days 6 and 14pi. This suggests an increase in adaptive T cells and a decrease in unconventional mucosal T cells in response to EHV-1 infection. Previous assessments of respiratory tract immune cells during EHV-1 infection focused on cells in lymphoid tissue or the LRT. Retropharyngeal and submandibular lymph nodes and nasopharyngeal tonsils were collected on day 7pi and supported the presence of EHV-1-specific cytolytic cells in lymphatic tissues of the URT [28], but access to these sites required euthanasia, preventing longitudinal analysis. While the involvement of the LRT in EHV-1 infection, replication, disease, and in the progression of the virus to cell-associated viremia is limited, it is physically connected to the URT, causing scientists to explore the LRT . Infectious virus has previously been detected in the trachea and lung of EHV-1-infected horses [12], which may explain the observed decrease in the CD4:CD8 ratio of bronchoalveolar lavage fluid cells by day 14pi in susceptible horses [82]. However, the role of LRT cells in a protective immune response is limited, and this population differs from the intranasal T cells that would first confront the virus. This has previously been supported in a mouse model of influenza infection, which identified differences in viral specificity of LRT versus URT mucosal T_RM_ cells, influenced by the environment in which they matured [83]. In vitro studies of T cells at the mucosa have primarily explored their role in viral pathogenesis. This has demonstrated that blood-derived T cells can be infected by cell-free EHV-1 or via cell-to-cell transfer from EHV-1-infected equine respiratory epithelial cells [31]. Similarly, ex vivo infection of nasal tissue resulted in EHV-1 infection of CD3^+^ T cells located beneath the basement membrane [84]. Together, these studies hypothesized that T cells could participate in the establishment of viremia, following T-cell infection at the mucosal surface, trafficking into the local lymphoid tissues, and spreading into the circulation. 

Other alphaherpesviruses, including herpes simplex virus 2 (HSV-2) and Varicella-Zoster Virus (VZV), attract virus-specific T cells to the site of primary infection, which are retained as T_RM_ cells in the vaginal mucosa and skin, respectively [62,63]. Here, we explored if nasal mucosal T cells can produce IFN-γ in response to EHV-1 re-stimulation. While mucosal T cells from healthy horses were generally able to produce IFN-γ after polyclonal stimulation through their TCR, EHV-1 re-stimulation did not result in mucosal IFN-γ^+^ T-cell detection, despite EHV-1-specific IFN-γ^+^ T cells in the peripheral blood of the same horses. In this study, low cell numbers limited further in vitro analysis of IFN-γ induction; however, characterization of other T-cell effector molecules, including cytokines and cytotoxic granules, is needed in future studies. The lack of EHV-1-specific T-cell responses in healthy horses can be due to several reasons: (1) EHV-1-specific T cells and/or T memory cells were not established in the URT after the last exposure with EHV-1, which was 30 or 72 months prior to this in vitro re-stimulation, and seem overall unlikely; (2) mucosal EHV-1-specific T cells live shorter than their circulating EHV-1-specific counterparts in the same horses; (3) in vaccinated horses, only systemic EHV-1-specific T cells are established and the cells are not trafficking to the mucosal URT tissue in healthy horses. Two out of five horses in the high responder group were never infected with EHV-1, only vaccinated; (4) the sampled mucosal cell populations lacked appropriate antigen-presenting cells to enable the activation of these cells after EHV-1 re-stimulation; (5) mucosal T cells respond to EHV-1 re-stimulation with responses other than IFN-γ; or (6) mucosal T cells taken out of the tissue context are anergic to the experimental conditions of the in vitro EHV-1 re-stimulation. In summary, the absence of a local EHV-1-specific T-cell population in healthy horses is interesting and requires additional experimental follow-up in future studies, for example, by evaluating mucosal immune cell functions in recently infected horses and/or by analyzing other T-cell responses, not just IFN-γ. 

## 5. Conclusions

The URT is the entry site for many respiratory viruses, including EHV-1. Nasal mucosal T-cell populations in horses have not been previously characterized. We showed here that CD4^+^ and CD8^+^ T cells represent the majority of immune cells in the nasal lumen and the respiratory mucosa. Although these cells are not available in large quantities when non or minorly invasive sampling procedures like nasal washes, swabs, or brushes are used in healthy horses, the abundance and prominent distribution of CD4^+^ and CD8^+^ T cells within the respiratory mucosa confirms their crucial role as gatekeepers at the mucosal surfaces of the URT. Towards the end of the first week of EHV-1 infection, T cells are attracted to the nasal cavity, intranasal T-cell numbers increase rapidly, and CD4^+^ and CD8^+^ T-cell proportions stay elevated even after the respiratory infection has cleared by two weeks pi. While the precise functional properties and subpopulations of mucosal T cells in horses still require additional characterization, these novel data highlight the substantial numbers of mucosal T cells present in the URT of healthy horses and provide new insights into T-cell dynamics during EHV-1 infection.

## Figures and Tables

**Table 1:** Clinical outcomes of EHV-1 infection.

Horse Information ^a^		Highest Temperature ^b^		Peak of Nasal Shedding ^c^		Peak of Viremia ^d^	
ID	Gender	°C	Hours Post-Infection	Copy # (gB/mL)	Day Post- Infection	Ct	Day Post- Infection
F1-2	colt	40.6	36	9.8 × 10^7^	2	32.064	6
F2-2	filly	40.0	48	1.2 × 10^8^	1	32.031	5
F3-2	filly	40.9	36	2.6 × 10^7^	1	27.901	9
F4-2	colt	40.0	36	8.8 × 10^7^	2	31.837	6
F5-2	colt	39.9	36	7.2 × 10^7^	2	33.570	7
F6-2	filly	39.7	36	1.7 × 10^7^	1	31.346	7
F7-2	colt	40.6	36	1.6 × 10^8^	1	31.665	6
F8-2	filly	40.3	36	2.4 × 10^7^	1	31.716	6
F9-2	colt	40.2	36	3.1 × 10^7^	2	30.237	6
F10-2	colt	40.6	36	2.2 × 10^8^	2	33.609	7
F11-2	filly	40.3	36	7.0 × 10^7^	2	31.881	6
F12-2	filly	40.4	36	2.8 × 10^7^	1	31.802	6
F13-2	colt	39.8	36	5.5 × 10^7^	1	33.229	6
F14-2	colt	40.3	36	3.9 × 10^7^	1	31.312	7
F15-2	colt	40.5	36	1.2 × 10^8^	2	31.806	6

^a^ The experimental infection study of these horses and all experimental details were previously described in detail (37). ^b^ Body temperatures measured by rectal temperature; fever was defined as body temperature of 38.6 °C or higher. ^c^ Nasal shedding and ^d^ cell-associated viremia were measured in PBMC by EHV-1-specific qPCR.

**Table 2:** Existing systemic T-cell immunity in healthy horses.

Horse Information				EHV-1 Specific T-Cell Response ^a^	
ID	Gender	Age (Years)	Last EHV-1 Exposure Prior to this Study (Months)	Classification of T-Cell Immunity	IFN-γ Secretion (MFI)
M11	mare	19	73 ^b^	IFN-γ low responder	620
F11-1	gelding	12	30 ^c^		1838
F1-2	gelding	11	30 ^d^		1
F20-8	gelding	5	30 ^c^		199
F3-21	gelding	2	never		1
M32	mare	10	73 ^b^	IFN-γ high responder	12,062
M33	mare	10	73 ^b^		16,204
F3-1	gelding	12	30 ^c^		14,027
F4-3	gelding	10	30 ^c^		8040
F16-7	gelding	6	30 ^d^		4593

^a^ Systemic T-cells responses were determined by IFN-γ secretion from the horse’s PBMC after re-stimulation with the EHV-1 strain Ab4 (MOI = 1) for 48 h, to classify the horses as low or high responders. IFN-γ was measured in the cell culture supernatants using the Equine Cytokine 5-plex assay. MFI = median fluorescence intensity. ^b^ Several times vaccinated with inactivated EHV or modified-live vaccine. ^c^ Exposed to EHV-1 infected horse. ^d^ Experimentally infected with EHV-1 Ab4.

## Data Availability

All data and information supporting the findings of this study are available within this article. Further inquiries can be directed to the corresponding author.
